# Insights from Femtosecond Transient Absorption Spectroscopy into the Structure–Function Relationship of Glyceline Deep Eutectic Solvents

**DOI:** 10.3390/molecules30051059

**Published:** 2025-02-26

**Authors:** Rathiesh Pandian, Clemens Burda

**Affiliations:** Department of Chemistry, College of Arts and Sciences, Case Western Reserve University, Cleveland, OH 44106, USA; rrp45@case.edu

**Keywords:** deep eutectic solvents, femtosecond transient absorption spectroscopy, solvation dynamics, solvation structure, glyceline, structure–function relationship, Reichardt’s dye betaine-30, *E*_T_(30) polarity

## Abstract

This study aimed to determine the structure–function relationship (SFR) for ChCl–glycerol mixtures, a deep eutectic solvent (DES), by investigating their microscopic solvation dynamics and how it relates to their macroscopic properties across varying concentrations of ChCl. Femtosecond transient absorption (fs-TA) spectroscopy revealed two distinct solvation dynamics time constants: *τ*_1_, governed by glycerol–glycerol interactions, and *τ*_2_, dominated by the choline response. The *τ*_2_ minimum at 25–30 mol % ChCl closely aligned with the eutectic composition (~33.33 mol % ChCl), where the glycerol network was the most organized and the choline ions exhibited the fastest relaxation. The viscosity decreased sharply up to ~25 mol % ChCl and then plateaued, while the conductivity increased monotonically with ChCl concentration, reflecting enhanced ionic mobility. The density decreased with both increasing ChCl concentration and temperature, indicating disrupted hydrogen bonding and reduced molecular packing. The polarity, measured using betaine-30 (B30) and the *E*_T_(30) polarity scale, increased steeply up to approximately 25 mol % ChCl before reaching a plateau. These findings identified the eutectic composition as the optimal concentration range for balancing stability, fluidity, conductivity, and enhanced dynamics within the glycerol system.

## 1. Introduction

Globally, the demand for energy is on the rise as the human population increases, resulting in an increased necessity to industrialize. This often entails the utilization of fossil fuels to match the energy demands. To ensure a sustainable future, developing and adopting renewable energy solutions is crucial. Renewable sources like wind, water, and solar are excellent alternatives to fossil fuels but are limited by intermittent and unpredictable availability. Reliable energy storage and efficient transport systems are essential to addressing these challenges. Rechargeable batteries, such as lithium-ion batteries, have revolutionized energy storage and transport but may not suffice for the large-scale electricity demands of the future. A robust and reliable large-scale battery system is necessary to support renewable energy integration and ensure sustainable energy distribution [1]. To sustain more grid-scale operations, redox flow batteries (RFBs) can contribute. Depending on their electrolyte composition, they can have high scalability, longevity, safety, biodegradability, and cost-effectiveness. They provide flexible, long-lasting discharge times, making them ideal for supporting long-term renewable energy storage [2,3,4].

Despite their promise, current RFBs face key challenges that limit their widespread use. Conventional aqueous RFB electrolyte solutions suffer from a narrow electrochemical window, restricting energy density [5]. A potential solution to this issue is to derive RFBs using electrolyte solvents, such as deep eutectic solvents (DESs). A DES is a type of liquid mixture formed by combining two or more components in varying proportions, typically involving a hydrogen bond acceptor (HBA) and a hydrogen bond donor (HBD), which interact through hydrogen bonding to create a eutectic mixture. This interaction significantly lowers the melting point of the mixture compared to the individual components, often resulting in a liquid at room temperature. They were discovered by Abbott et al. in 2004, who found that mixtures consisting of choline chloride (ChCl) as the HBA and urea as the HBD resulted in mixtures with lower melting points than their individual components [6,7]. DESs are considered environmentally friendly alternatives to conventional solvents due to their low toxicity, biodegradability, and ease of preparation. They can be chemically and thermally stable, enabling operation under diverse environmental conditions [8,9]. Their physical and chemical properties are tunable by varying the HBA, HBD, and/or their mixing proportions, making them useful electrolytes in RFBs [1,2,10,11,12,13].

In the past two decades, numerous DES systems have been explored, including reline (1 mol ChCl + 2 mol urea), ethaline (1 mol ChCl + 2 mol ethylene glycol), and glyceline (1 mol ChCl + 2 mol glycerol) [7,11,14,15,16,17,18,19,20,21], to name a few. Some other interesting parameters that have been explored are the effects of co-solvent/water addition [22,23,24,25,26,27,28,29,30,31,32], HBA variation [27,30,33,34,35,36,37], and HBD variation [38,39,40,41,42]. The properties of DESs, are primarily dependent on hydrogen bonding. The complex hydrogen bonding network in DESs significantly impacts their physical and chemical properties, including viscosity, melting point, density, dynamics, and charge transport [18,22,38,43,44,45]. Glycerol-based DESs are of particular interest due to their high hydrogen-bonding capability and the ability to module their viscosity by varying the ChCl content [15]. Although there has been extensive research into DESs, fundamental uncertainties persist regarding the connection between their microscopic solvation dynamics and macroscopic transport properties.

**Scheme 1:**
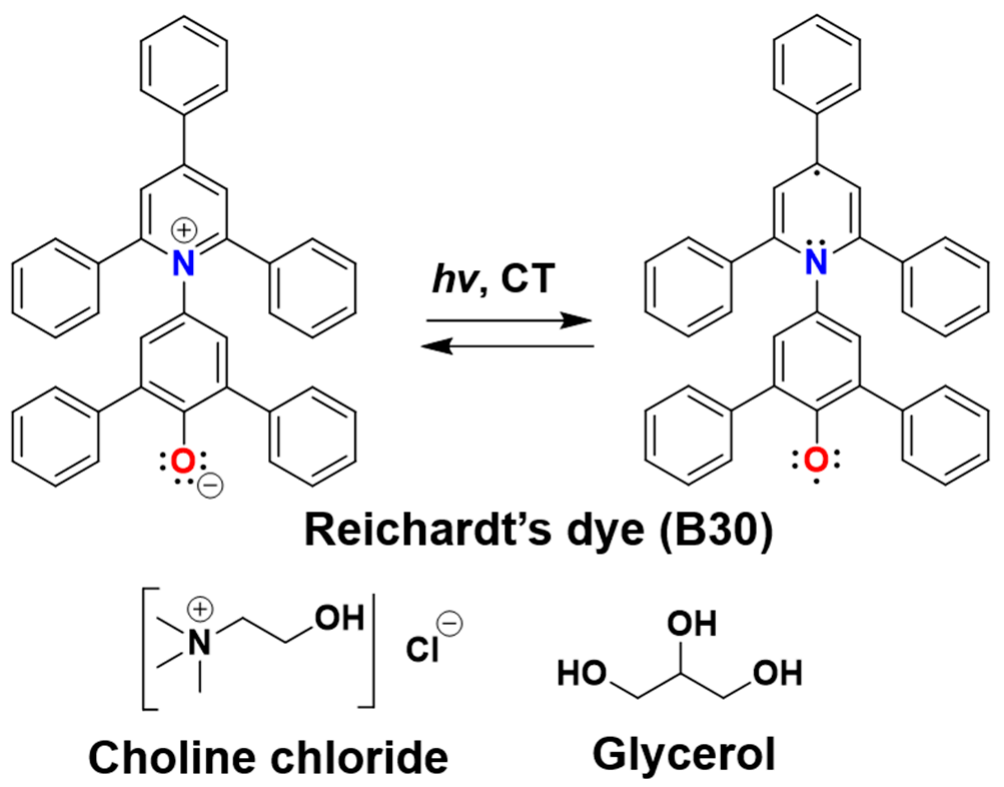
Representative molecular structures for the laser pulse-induced intramolecular charge transfer process that occurs in B30 upon its photoexcitation from its Zwitterionic ground state to its radicalized excited state, depicted from left to right. The structures of ChCl and glycerol are also shown.

To address these knowledge gaps, we investigated the solvation dynamics of ChCl–glycerol mixtures with varying mol % ChCl concentrations. The goal was to study the solvation dynamics of glycerol-based DESs to ascertain if there is a structure–function relationship (SFR) between their microscopic solvation and their properties such as viscosity, ionic conductivity, and density [26,27,38,46]. The solvation dynamics were investigated by probing the solvent relaxation response to a photoexcited probe molecule, Reichardt’s dye betaine-30 (B30), developed by Christian Reichardt in 1963 [47], which was dissolved in the DES systems. Upon photoexcitation, B30 undergoes an essentially instantaneous [48,49] intramolecular charge transfer (CT), where the electron density shifts from the phenolate group to the pyridinium ring, resulting in a significant change in B30’s dipole moment [50,51,52]. The observed back-electron transfer kinetics are governed by the solvent reorganization response and not by the probe molecule itself. The solvent reorganization process is the slower, rate-limiting step in this mechanism, and the dynamics vary depending on the surrounding solvent environment and not on the probe itself [48,49]. As such, B30 serves as a probe molecule, with its response providing a direct measure of solvent reorganization in DES systems, making it a valuable tool for investigating solvation dynamics in ChCl–glycerol mixtures. In addition to dynamics, polarity was measured using the *E*_T_(30) polarity scale, a method that was established by Reichardt [45,47,53,54,55]. This polarity measurement takes advantage of the strong negative solvatochromism of B30. In a more polar system, the UV-Vis absorption band corresponding to the CT process will be further blue-shifted. This is due to the difference in dipole moment of B30’s zwitterionic ground state (+15 Debye) and excited radical state (−6 Debye) [56] (see Scheme 1). The ground state of B30 is more dipolar than its excited state, which makes it more effectively stabilized in polar solvents. As a result, in more polar solvent environments, B30 requires higher-energy light for photoexcitation. This leads to the observed blueshift in its CT absorption band, as described in a recent article on the history and functionality of B30 [45].

Recently, B30 has been employed as a solvatochromic probe to investigate solvation dynamics in various DES mixtures using femtosecond transient absorption (fs-TA) spectroscopy. In ChCl–ethylene glycol systems, Spittle et al. observed that the solvation dynamics were fastest near the eutectic composition (~20 mol % ChCl), a trend that also correlated with enhancements in ionic conductivity [46]. Similarly, Pandian et al. reported that in ChCl–propanediol systems, faster solvation dynamics coincided with increased ionic conductivity [38]. Beyond composition-dependent trends, solvation dynamics have also been explored in relation to other key factors, such as the impact of water addition in ethaline [26] and the effects of halide variation [27,30,33], both of which significantly influence conductivity and other crucial transport properties. While these studies have provided valuable insights into solvation dynamics in DESs, glycerol-based systems remain less explored despite their distinct hydrogen-bonding capabilities and widespread applicability.

In this study, we investigated glycerol-based DESs with varying molar percentages of choline chloride (ChCl). Unlike other HBDs such as ethylene glycol and other diols, glycerol is a triol. The presence of three -OH groups in glycerol means that it has the capability of forming a highly extended hydrogen-bonding network, which significantly impacts some of its properties [57]. As a pure solvent, glycerol molecules can form many hydrogen bonds, with intermolecular hydrogen-bonding being more prevalent than intramolecular ones. Molecular dynamics simulations by Root et al. estimated that in a cluster of 32 glycerol molecules, there would be 99 intermolecular and 5 intramolecular hydrogen bonds, which translates to approximately 3.09 intermolecular and 0.16 intramolecular hydrogen bonds per glycerol molecule [58]. A later study by Zhuang et al. found, through ab initio methods, that each glycerol molecule donates 5.7 intermolecular and 0.8 intramolecular hydrogen bonds [59]. This is in good agreement with the findings of Padró et al., who reported 5.7 hydrogen bonds per glycerol molecule through molecular dynamics simulations, and also noted that ethylene glycol (a diol) forms 3.9 hydrogen bonds per molecule, while mono-alcohols like methanol and ethanol form 1.9 hydrogen bonds per molecule [60]. More recently, Towey et al., combining neutron diffraction with isotopic substitution and empirical potential structure refinement modeling, found that each glycerol molecule donates 5.68 ± 1.51 intermolecular hydrogen bonds, with no evidence of the presence of intramolecular hydrogen bonds [61]. Together, these studies confirm that the hydrogen-bonding network in glycerol is extensive.

The introduction of ChCl into glycerol’s extended hydrogen-bonding network is expected to strongly influence the solvation dynamics, viscosity, ionic conductivity, and density. When ChCl is added, glycerol exhibits stronger hydrogen-bonding interactions with chloride compared to choline. However, despite the increased hydrogen bonding with chloride, intermolecular glycerol–glycerol hydrogen bonding interactions are still dominant, even in as much as 33.33 mol % ChCl [57,62]. While previous studies have highlighted the role of hydrogen bonding in shaping DES properties, the direct link between microscopic solvation dynamics and bulk transport properties remains underexplored in glycerol-based DESs. Using fs-TA spectroscopy, we aimed to better understand how ChCl incorporation affects glycerol’s solvation environment and impacts its functional properties. Establishing these correlations will not only improve our understanding of glycerol-based DESs but also define a clear SFR between solvation dynamics and macroscopic transport properties, offering valuable insight into glycerol-based DESs’ viability as next-generation electrolytes for energy-storage applications. We focused our measurements on 0–33.33 mol % ChCl because our primary focus was to correlate solvation dynamics around the eutectic composition, measuring the trends in conductivity and viscosity in this range of concentrations. Simulating ChCl–glycerol mixtures presents significant challenges due to the complex hydrogen-bonding network between choline, chloride, and glycerol molecules. Therefore, this study focused solely on experimental results to provide insights into the system’s structural and dynamic properties.

## 2. Results

### 2.1. Solvent Relaxation Dynamics

Fs-TA measurements were used to investigate the solvent relaxation dynamics following laser-induced CT. The time constants for each system were determined by biexponentially fitting the experimentally obtained kinetic decay traces using Equation (1).

TA(*t*, *λ*) = *A*_1_ exp(−*t*/*τ*_1_) + *A*_2_ exp(−*t*/*τ*_2_) (1)

The fitting parameters included the amplitudes (*A*_1_ and *A*_2_) and time constants (*τ*_1_ and *τ*_2_), with R^2^ values exceeding 0.99. The response of solvent molecules to the electronic rearrangement of B30 follows a biexponential pattern, where the faster time constant, *τ*_1_, corresponds to the local solvent response on the picosecond timescale, while the slower component, *τ*_2_, captures the complex interactions within the DES hydrogen-bond network [63]. While B30 undergoes a CT upon excitation, the presented analysis focuses on the resulting relaxation dynamics of the ChCl–glycerol system. In the case of these glycerol-based mixtures, *τ*_1_ is likely driven by dynamics dominated by the glycerol–glycerol hydrogen bonding network. *τ*_2_ is primarily due to choline because in choline halide-based DESs, self-diffusion measurements have revealed that choline is the slowest component in the mixture [11,27,64].

**Figure 1:**
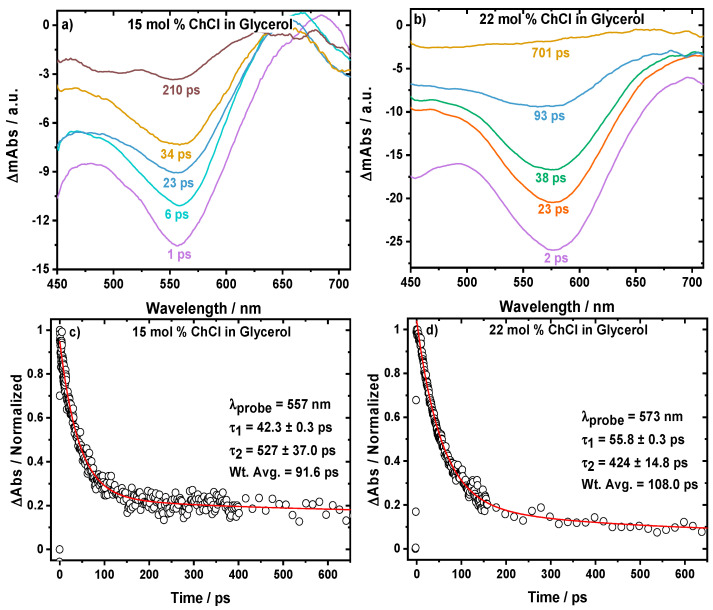
Sample fs-TA spectra of B30 dissolved in (**a**) 15 mol % and (**b**) 22 mol % ChCl in glycerol. The negative signal primarily corresponds to ground-state bleaching. As the delay time increases, the signal becomes less negative, which shows the recovery of the ground state. The corresponding biexponential kinetic decay traces are shown in panels (**c**,**d**), respectively. Fitting was performed in the spectral range of *λ*_fitted_ = 500–600 nm. *T* = 298 K.

Sample transient spectra are shown in Figure 1a,b at 15 and 22 mol % ChCl, respectively. The negative signal was due to the spectral feature of ground-state bleaching, which is most negative right after photoexcitation. The subsequent recovery of the ground state is depicted by the transient bleaching signal becoming less negative as a function of increasing delay time [65]. From the transient spectra, a kinetic decay trace can be derived, which is shown in Figure 1c,d. Equation (1) was used to biexponentially fit the traces to determine the time constants.

**Figure 2:**
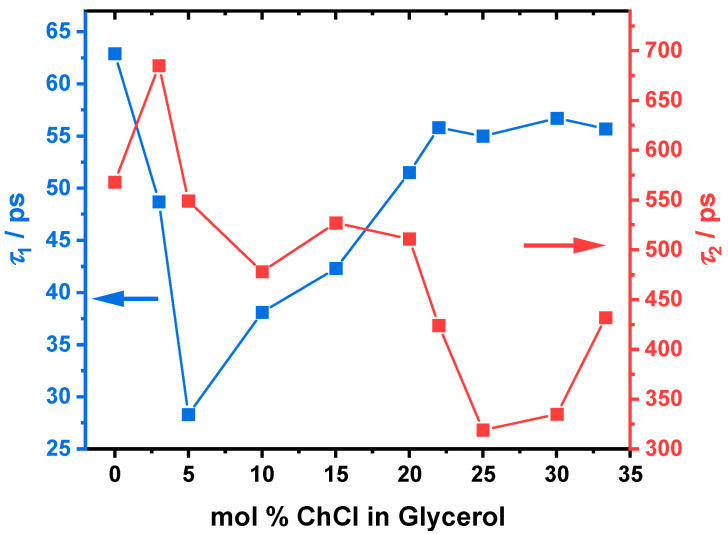
Fast (*τ*_1_; blue y-axis on left) and slow (*τ*_2_; red y-axis on right) solvent relaxation components for the ChCl–glycerol mixture as a function of mol % ChCl. There is a clear minimum in *τ*_1_ at ~5 mol % ChCl, followed by an increase and plateauing after ~20–25 mol % ChCl. For *τ*_2_, there are two distinct minima, with the slower one at ~10 mol % ChCl and the faster one at ~25–30 mol % ChCl. The kinetics data were acquired from fitting the transient absorption spectra at *λ*_fitted_ = 500–600 nm. See Table S1 for numerical values. *T* = 298 K.

The parameters *τ*_1_ and *τ*_2_ are dependent on the composition of the mixtures and are portrayed in Figure 2. Regarding the faster component *τ*_1_, the dynamics were fastest at lower salt concentrations, reaching a minimum of 28.3 ps at 5 mol % ChCl. Following this, the *τ*_1_ dynamics slowed down/increased in value, reaching a plateau at ~20–25 mol % ChCl and it hovered around ~55 ps. For the slower component *τ*_2_, there appeared to be two minima: a slower one at ~10 mol % ChCl (~700 ps) and another faster one at 25–30 mol % ChCl (~450 ps).

### 2.2. Viscosity, Ionic Conductivity, and Density Measurements

**Figure 3:**
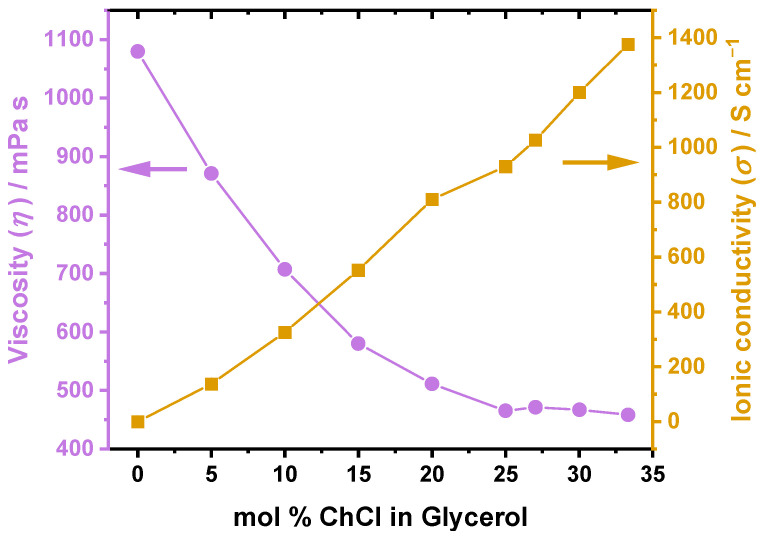
Viscosity (left y-axis) and ionic conductivity (right y-axis) as a function of mol % ChCl in glycerol. Viscosity decreases as ChCl is added, until it plateaus after ~25 mol ChCl. Conductivity increases monotonically throughout the entire composition range. See Table S2 for numerical values and standard deviations. N = 3, *T* = 298 K.

**Figure 4:**
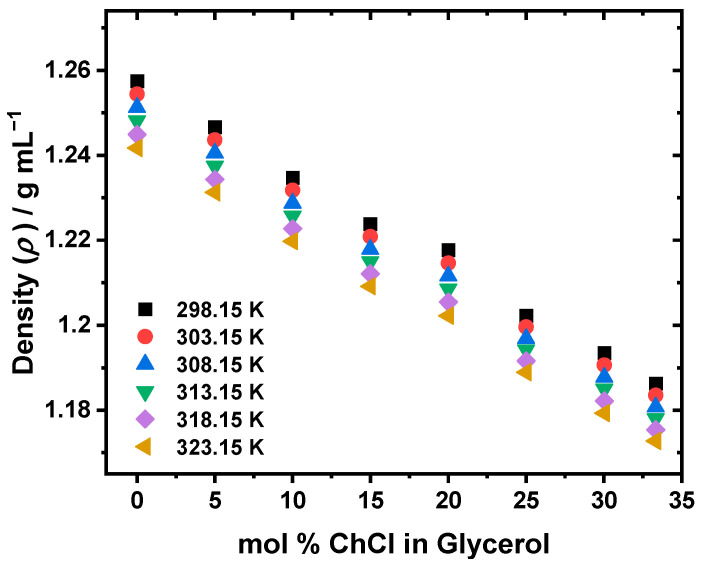
Density of glyceline as a function of mol % ChCl in glycerol. The legend depicts the data points representing the various temperatures from 298.15 to 323.15 K in 5 K increments. See Table S3 for numerical values and standard deviations. See Figure S1 for density represented as a function of temperature. N = 3.

The viscosity (*η*), ionic conductivity (*σ*), and density (*ρ*) of the ChCl–glycerol solvent systems with varying mol % ChCl concentrations were measured. The left y-axis of Figure 3 shows the viscosity of the mixture as a function of increasing mol % ChCl, which was monoexponential in nature. As ChCl was added, the viscosity decreased sharply until ~25 mol % ChCl, after which, the viscosity plateaued at ~450 mPa s for the remaining concentrations. The ionic conductivity, represented on the right y-axis of Figure 3, increased monotonically with rising salt concentrations. The density of the mixtures decreased as a function of increasing mol % ChCl, as depicted in Figure 4. When the density was represented as a function of temperature (Figure S1), it decreased as a function of increasing temperature. The viscosity, conductivity, and density results reported in this work are in good agreement with values documented in the literature for ChCl–glycerol systems [15,16,66,67].

### 2.3. Solvent Polarity

To determine the solvent polarity, the *E*_T_(30) polarity scale, as established by Reichardt, was used. Determination of *E*_T_(30) was performed using Equation (2) [45,51,55].

*E*_T_(30) (kcal mol^−1^) = *h c N*_A_ *ν*_max_ = 28591/*λ*_max_ (2)

**Figure 5:**
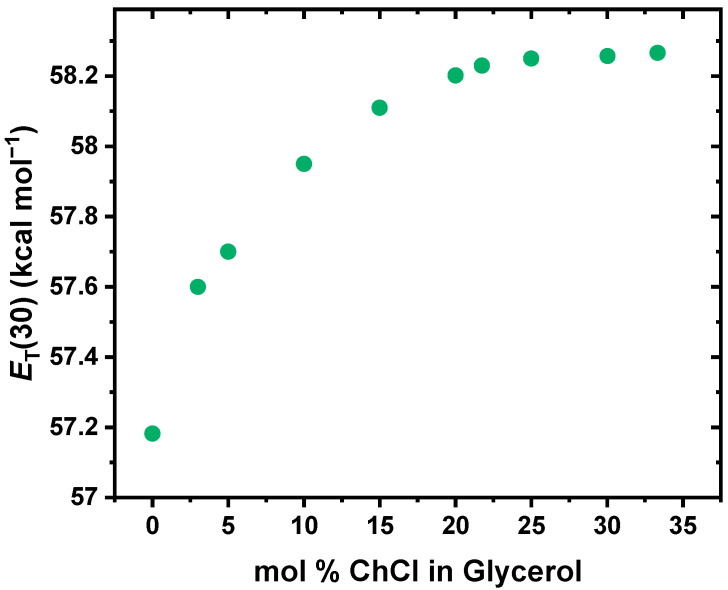
*E*_T_(30) polarity versus mol % ChCl in glycerol. The increase in polarity plateaus at >25 mol % ChCl. See Table S1 for numerical values. *T* = 298 K.

In this equation, *h* represents the Planck constant, *c* is the speed of light, *N*_A_ is Avogadro’s number, and *ν*_max_ and *λ*_max_ correspond to the wavenumber and wavelength, respectively, at the absorption maximum of B30 for its longest-wavelength CT transition (Scheme 1). B30 is negatively solvatochromic, exhibiting a blueshift in the UV-Vis absorbance of the CT band as the solvent system’s polarity increases [26,65,68]. Using Equation (2), a blueshift in the *λ*_max_ corresponds to higher *E*_T_(30) values; thus, a greater *E*_T_(30) denotes a solvent system that is more polar. From Figure 5, the *E*_T_(30) polarity increased monotonously as the salt concentration increased. As small quantities of ChCl were added (0–10 mol % ChCl), the polarity rose sharply, but then reached a plateau when the ChCl concentrations exceed 25 mol %. This increase in polarity is in good agreement with recent work on similar systems [38].

## 3. Discussion

The dominance of the glycerol hydrogen-bonding network in glyceline (33.33 mol % ChCl in glycerol) is particularly evident at the eutectic composition, which is at ~33.33 mol % ChCl, according to the work from Abbott et al. [15]. The glycerol hydrogen-bonding network forms a structured framework, similar to that of pure glycerol, where each glycerol molecule forms an average of about 5.7 hydrogen bonds [60,61]. This extensive hydrogen-bonding network remains largely intact at low ChCl concentrations, where glycerol’s network continues to dominate the solvent’s structure [57,62]. The H-bond network serves as the structural framework of the solvent, while choline cations act as a loosely bound species occupying interstitial voids with minimal direct interactions with the glycerol hydrogen-bonding network [62,69]. Upon the addition of ChCl, glycerol–glycerol hydrogen bonds are disrupted as chloride ions compete for hydrogen bonding sites. This results in increased chloride ion coordination with glycerol, integrating chloride into the hydrogen-bonding network [57,69]. The solvation dynamics of ChCl–glycerol mixtures provide valuable insights into the interplay between structural and dynamic behaviors, particularly regarding the role of choline ions. The Fs-TA spectroscopy measurements (sample spectra and kinetics illustrated in Figure 1) reveal two distinct time constants derived from biexponential fitting of the kinetic decay curves (Figure 2). We associated these time constants as follows: *τ*_1_ (the faster component) was associated with local solvation dynamics dominated by glycerol–glycerol hydrogen bonding, while *τ*_2_ (the slower component) was linked to the bulk solvent dynamics primarily governed by the response of choline ions. The assignment of *τ*_1_ to the glycerol hydrogen-bonding network is supported by prior studies on solvation dynamics in DESs. Wagle et al. demonstrated through quasielastic neutron scattering that in 1:2 ChCl–glycerol mixtures, glycerol forms stronger hydrogen bonds with chloride than with choline, leading to an extended hydrogen-bonding network [70]. This may suggest that the solvation dynamics are predominantly governed by glycerol–glycerol and glycerol–chloride interactions, with choline playing a more limited role. While both glycerol–glycerol and glycerol–chloride interactions contribute to solvation dynamics, the glycerol–glycerol interactions are still the most dominant ones, even in as much as 33.33 mol % ChCl, as shown by Turner et al. [57]. Additional support for this assignment comes from self-diffusion measurements of glyceline. D’Agostino et al. reported that at 298 K, glycerol diffuses faster (0.52 × 10^11^ m^2^ s^−1^) than choline (0.38 × 10^11^ m^2^ s^−1^) [64]. Given that *τ*_1_ corresponds to the faster relaxation component, it is reasonable to attribute it to solvent reorganization dominated by the glycerol hydrogen-bonding network. Conversely, *τ*_2_ is attributed to the slower choline-associated response. This interpretation is consistent with what is measured in the similar ChCl–ethylene glycol-based systems, where *τ*_1_ occurs on the order of tens of picoseconds, while *τ*_2_ is on the order of hundreds of picoseconds [26,46].

Notably, *τ*_1_ initially reached a minimum at 5 mol % ChCl, thereafter steadily increasing and plateauing at 20–25 mol % ChCl. This upward trend suggests that glycerol hydrogen-bonding re-establishes a more organized network as the solvent approaches the eutectic composition. For *τ*_2_, two minima were observed: a slower one at ~10 mol % ChCl and a faster one at 25–30 mol % ChCl. The presence of two minima in *τ*_2_ seems to be unique to the ChCl–glycerol system, as this was not present in studies performed on diols [38,46]. This may be due to the highly extended hydrogen-bonding network of glycerol [57] or the heterogeneous nature of these mixtures [17]. The faster of the two minima in *τ*_2_ between 25 and 30 mol % ChCl closely aligns with the eutectic composition of glyceline (~33.33 mol % ChCl) [15,17]. This *τ*_2_ minimum indicates that choline ions attain their fastest relaxation dynamics in this concentration range where choline is most effectively integrated into the glycerol network. Based on the literature [62], we concluded that this integration minimizes the constraints on choline mobility, enabling its faster *τ*_2_ relaxation. The interplay between solvent structure and dynamics highlights a novel form of a structure–function relationship, wherein glycerol predominates in the structural organization of the solvent, providing robustness and stability, while the choline ions that are integrated into the glycerol network retain sufficient mobility to enhance the system’s fluidity and conductivity at higher salt concentrations.

Regarding viscosity (Figure 3, left y-axis), there was a marked decrease until 20–25 mol % ChCl, after which, the viscosity decrease became less pronounced to the point where it plateaued at around the eutectic composition. This phenomenon may be elucidated by the *τ*_1_ dynamics. As previously mentioned, *τ*_1_ decreased initially from 3 to 5 mol %, and then increased steadily until plateauing at 20–25 mol % ChCl. Similarly, the viscosity decreased steadily until it reached a plateau at a similar ChCl concentration. The initial decrease in *τ*_1_ reflects localized disruption of the glycerol–glycerol hydrogen-bonding network as chloride anions begin to coordinate with glycerol molecules [16]. However, this disruption was not extensive, as the bulk hydrogen-bonding framework remained intact [62]. As the ChCl concentration increased beyond 5 mol %, the glycerol network may have reorganized to incorporate ionic species more effectively, leading to a steady rise in *τ*_1_ and its eventual stabilization at 20 mol %. Despite the reorganization of the glycerol hydrogen-bonding network becoming more efficient with increasing ChCl concentrations, there was still the chloride disrupting a small number of glycerol–glycerol interactions, resulting in the decrease in viscosity that was measured. In terms of conductivity (Figure 3, right y-axis), the conductivity increased almost linearly as a function of increasing mol % ChCl. This increase may be attributed to the augmentation in the number of charged species present in the mixtures concomitant with the addition of ChCl [16]. The observed enhancement in conductivity with increasing ChCl concentration suggests that chloride anions remain relatively free and are not significantly restricted by interactions with glycerol [57]. The solvation dynamics, particularly the *τ*_2_ data, revealed a correlation between the fastest relaxation dynamics and the eutectic composition. This solvent structure–function relationship directly aligns with the enhancements in viscosity and conductivity, reflecting optimized structural and dynamic properties near the eutectic composition. Similar trends have recently been reported for ethaline [38,46].

The density (Figure 4) of the ChCl–glycerol system decreased with both increasing ChCl concentration and rising temperature. The nearly linear decline in density with ChCl concentration reflects the progressive disruption of glycerol’s dense hydrogen-bonding network due to the addition of ChCl. While the majority of the extended glycerol–glycerol hydrogen bond network remains intact upon ChCl addition, a small number of those interactions are disrupted as glycerol coordinates with chloride. The incorporation of chloride into this network results in a less compact structure, thus lowering density as the mol % of ChCl increases [37,71]. Similarly, the density decreases with increasing temperature due to thermal expansion and the formation of larger intermolecular voids, which also reduces molecular packing [72].

To assess polarity (Figure 5), the *E*_T_(30) polarity scale was employed, wherein higher values correspond to more polar solvent environments [45,54,73]. The polarity increased with rising ChCl concentrations, which aligns with the trends from other solvent systems [38]. This indicates that the polarity and structure of the solvent system is indeed sensitive to ChCl addition, but only to a certain point. Up to 20–25 mol % ChCl, the increase in polarity was steep, which also coincided with a steep decrease in viscosity and density. This phenomenon may be attributed to the initial disruption of the glycerol–glycerol network with small quantities of ChCl (5 mol % ChCl), leading to the formation of some glycerol–chloride hydrogen bonding. As the amount of ChCl increases, the system becomes more ionic in character, enhancing its capacity to stabilize the charge transfer (CT) state of B30. At the higher mol % ChCl region (>25 mol % ChCl), the glycerol hydrogen-bonding network is re-established resulting in the plateauing of *E*_T_(30) polarity at approximately the eutectic composition. Additionally, this plateau reflects the saturation of ionic contributions to polarity, as the glyceline system contains sufficient ions to saturate and maximize polarity.

## 4. Materials and Methods

### 4.1. Materials

Choline chloride (ChCl) (≥98%), glycerol (≥99.5%), and Reichardt’s dye betaine-30 (B30) were all purchased from Sigma-Aldrich (St. Louis, MO, USA). Scheme 1 depicts the chemicals used in this work.

### 4.2. Preparation of Solvent Mixtures

The ChCl was dried under a vacuum at 120 °C for 72 h and then stored in a glove box under an argon atmosphere. The glycerol was dried using molecular sieves. For sample preparation, ChCl was weighed in the glove box using an analytical balance (±0.0001 g precision), which was securely sealed to prevent moisture absorption, and then removed from the glove box. A pre-measured quantity of the dried glycerol was added to the ChCl, and the resulting mixture was sonicated with heating for several hours to yield a stock mixture containing 33.33 mol % ChCl in glycerol. From this stock, dilutions were then prepared by mixing calculated amounts of the stock solution with pure glycerol to obtain mixtures with varying mol % ChCl concentrations. For the 0% ChCl sample, only pure glycerol was used without the addition of ChCl. Prior to conducting the measurements, the utmost care was taken to ensure that the samples were sonicated to minimize any heterogeneity. In our experience, sample preparation must be performed in a consistent manner to ensure consistency in the measurements.

### 4.3. Measurements of Relaxation Dynamics and Solvent Polarity

To measure relaxation dynamics and polarity, a small amount of B30 dye was dissolved into each mixture until an optical density of 0.5 per 0.2 cm optical path length was achieved. A Varian (Santa Clara, CA, USA) Cary 50 UV-Vis spectrophotometer was used to collect steady-state absorption spectra of these B30-enriched mixtures, which had a maximum error of ±1 nm. The position of the CT absorption band maximum was solvatochromic, varying with the solvent. Solvent polarity was assessed using the *E*_T_(30) scale, as developed by Reichardt, which capitalizes on the strong solvatochromism of B30 [51,55,68,73,74]. To measure the solvent relaxation and kinetics, femtosecond transient absorption (fs-TA) spectroscopy was performed using B30, as established by Barbara in the 1990s [48,75]. The fs-TA experiment was conducted in triplicate using a Clark MXR (Dexter, MI, USA) CPA-2010 laser system (Ti:Sapphire regenerative amplifier, 780 nm fundamental beam, 130 fs pulse duration, 1 kHz repetition rate, and 850 mW output). The laser beam was split into two pathways: one was frequency-doubled to 390 nm with a *β*-barium borate crystal for the pump beam, while the other generated a broadband white-light supercontinuum (450–800 nm) with a sapphire crystal for the probe beam. A computer-controlled optical delay stage was employed to introduce delays for measuring the differential absorption spectra. Both steady-state and fs-TA measurements were performed in a 2 mm glass cuvette at room temperature and ambient pressure conditions. Steady-state absorption spectra, collected before and after the fs-TA experiment, confirmed that no photodegradation of B30 occurred. Optical chirp correction was performed using Surface Xplorer software (version 4.3.0), and biexponential fitting and data analysis were carried out using Microcal Origin (version 9.7.5.184); all the errors were less than ± 10%.

### 4.4. Measurements of Viscosity and Density

The viscosity (*η*) of the mixtures was determined by introducing approximately 100 µL of the sample into a Rheosense (San Ramon, CA, USA) microVISC viscometer in a temperature-controlled environment at 298 K. The resulting viscosity measurements reported in this work were collected in triplicate and averaged, with the uncertainties provided. The density (*ρ*) was measured using an Anton-Paar (Graz, Styria, Austria) DMA-5000 density meter at 5 K increments ranging from 298.15 to 323.15 K with a standard deviation ≤ ±0.5 K. The density meter was calibrated using degassed deionized water. The maximum density deviation at 293 K was determined to be 5 × 10^−5^ g mL^−1^.

### 4.5. Measurements of Ionic Conductivity

The ionic conductivity (*σ*) was measured using a Thermo Scientific (Waltham, MA, USA) Orion Star A222 conductivity meter with a 2-electrode conductivity cell that has a measurement range of 10 to 2000 mS cm^−1^ in a temperature-controlled environment at 298 K. Three standard sodium chloride solutions with conductivities of 0.100, 1.413, and 12.9 mS cm^−1^ were used to calibrate the instrument. The resulting conductivity measurements reported in this work were collected in triplicate, and the results were averaged, with the uncertainties provided.

## 5. Conclusions

This study explores the structure–function relationship between solvent composition and solvent dynamics in mixtures of ChCl and glycerol by examining their time-resolved solvation dynamics at the molecular level and how these relate to macroscopic transport properties such as viscosity, conductivity, and density. The eutectic composition of glyceline (~33.33 mol % ChCl) represents an optimal balance between structure and dynamics. Near this composition, the hydrogen-bonding network is highly organized, choline ions exhibit their fastest relaxation dynamics (*τ*_2_ minimum), and the viscosity stabilizes due to the effective incorporation of ions into the glycerol–glycerol network. The conductivity increases monotonically with ChCl concentration, attributed to the high chloride mobility, while the density decreases with ChCl addition and temperature, reflecting disrupted molecular packing. The polarity, as indicated by *E*_T_(30), reaches a plateau near the eutectic composition as the solvent system becomes optimized. These findings highlight the eutectic composition as a critical concentration for achieving an optimal balance of stability, fluidity, conductivity, and, in general, enhanced dynamic solvent properties in eutectic electrolyte systems.

## Data Availability

The original contributions presented in this study are included in the article/Supplementary Materials. Further inquiries can be directed to the corresponding author.

## Supplementary Materials

The following supporting information can be downloaded at: https://www.mdpi.com/article/10.3390/molecules30051059/s1, Figure S1: Density as a function of temperature (295.15–323.15 K), with legend representing varying mol % ChCl concentrations in glycerol solutions; Table S1: Maximum CT band absorption wavelength (*λ*_max_^abs^), *E*_T_(30) polarity, and dynamics (*τ*_1_, fast; *τ*_2_, slow; *τ*_avg_, average of the two) measurements for ChCl–glycerol systems with various mol % ChCl at 298 K; Table S2: Numerical values of viscosity (*η*) and ionic conductivity (*σ*) of ChCl–glycerol mixtures with varying mol % ChCl concentrations at 298 K; Table S3: Densities (*ρ*) of ChCl–glycerol mixtures with varying mol % ChCl concentrations at different temperatures.
