# Supplementary material for: Insights from Femtosecond Transient Absorption Spectroscopy into the Structure–Function Relationship of Glyceline Deep Eutectic Solvents
Source: Molecules. 2025 Feb 26;30(5):1059. doi: 10.3390/molecules30051059 (PMC11901628; doi:10.3390/molecules30051059)
Supplement: Supplementary file 1 [file molecules-30-01059-s001.zip › molecules-3469151-supplementary.pdf]

Supplementary Information

Insights from Femtosecond Transient Absorption Spectroscopy into the Structure-Function Relationship of Glyceline Deep Eutectic Solvents

Rathiesh Pandian and Clemens Burda *

Department of Chemistry, College of Arts and Sciences, Case Western Reserve University, Cleveland, OH 44106, USA; rrp45@case.edu
* Correspondence: burda@case.edu

Table S1. Maximum CT band absorption wavelength (λ_{max}^{abs}), $E_T(30)$ polarity, and dynamics (τ_1, fast; τ_2, slow; τ_{avg}, average of the two) measurements for ChCl:glycerol systems at various mol % ChCl. T = 298 K. N = 3.

mol % ChCl	λ_{max} (nm)	$E_T(30)$ (kcal mol^{-1})	τ_1(ps)	A_1	τ_2 (ps)	A_2	τ_{avg} (ps)[a]
0.00	500.00	57.18	62.9 ± 0.7	0.632	568 ± 19.3	0.234	199
3.00	496.37	57.60	48.7 ± 0.1	0.630	685 ± 7.76	0.210	208
5.00	495.51	57.70	28.3 ± 1.0	0.683	542 ± 3.58	0.161	126
10.00	493.37	57.95	38.1 ± 0.1	0.760	478 ± 4.72	0.207	132
15.00	492.02	58.11	42.3 ± 0.3	0.756	527 ± 37.0	0.086	91.6
20.00	491.24	58.20	51.5 ± 0.1	0.731	511 ± 9.47	0.186	145
22.00	491.00	58.23	55.8 ± 0.3	0.778	424 ± 14.8	0.128	108
25.00	490.83	58.25	55.0 ± 1.4	0.567	319 ± 21.3	0.219	129
30.00	490.77	58.26	56.7 ± 0.5	0.712	335 ± 31.0	0.168	110
33.33	490.70	58.27	55.7 ± 2.3	0.766	432 ± 29.2	0.181	128

[a] $\tau_{avg} = (A_1\,\tau_1 + A_2\,\tau_2)\,(A_1 + A_2)^{-1}$

Table S2. Numerical values of measured viscosity (η) and ionic conductivity (σ) of ChCl:glycerol mixtures at varying mol % ChCl with their standard deviation. *N* = 3. *T* = 298 K.

mol % ChCl	η (mPa s)	σ (S cm^{-1})
0.00	1079.7 ± 2.6	0.0 ± 0.0
5.00	871.0 ± 0.5	137.3 ± 0.5
10.00	707.0 ± 0.6	325.5 ± 1.1
15.00	580.1 ± 0.2	552.0 ± 3.4
20.00	511.4 ± 3.8	810.7 ± 0.7
25.00	465.4 ± 0.7	930.2 ± 13
27.00	471.2 ± 5.5	1027 ± 7.3
30.00	467.1 ± 2.3	1201 ± 2.9
33.33	458.4 ± 2.4	1376 ± 0.8

Table S3. Densities (ρ) of ChCl:glycerol mixtures at varying mol% ChCl and temperatures. *N* = 3.

mol % ChCl	ρ (g mL^{-1})					
	298.15 K	303.15 K	308.15 K	313.15 K	318.15 K	323.15 K
0.00	1.25748	1.25440	1.25127	1.24811	1.24489	1.24171
5.00	1.24665	1.24361	1.24054	1.23743	1.23429	1.23128
10.00	1.23471	1.23176	1.22874	1.22568	1.22273	1.21976
15.00	1.22380	1.22086	1.21789	1.21495	1.21208	1.20916
20.00	1.21765	1.21464	1.21160	1.20852	1.20544	1.20220
25.00	1.20227	1.19959	1.19686	1.19421	1.19157	1.18893
30.00	1.19347	1.19063	1.18778	1.18498	1.18218	1.17933
33.33	1.18628	1.18353	1.18076	1.17806	1.17540	1.17273

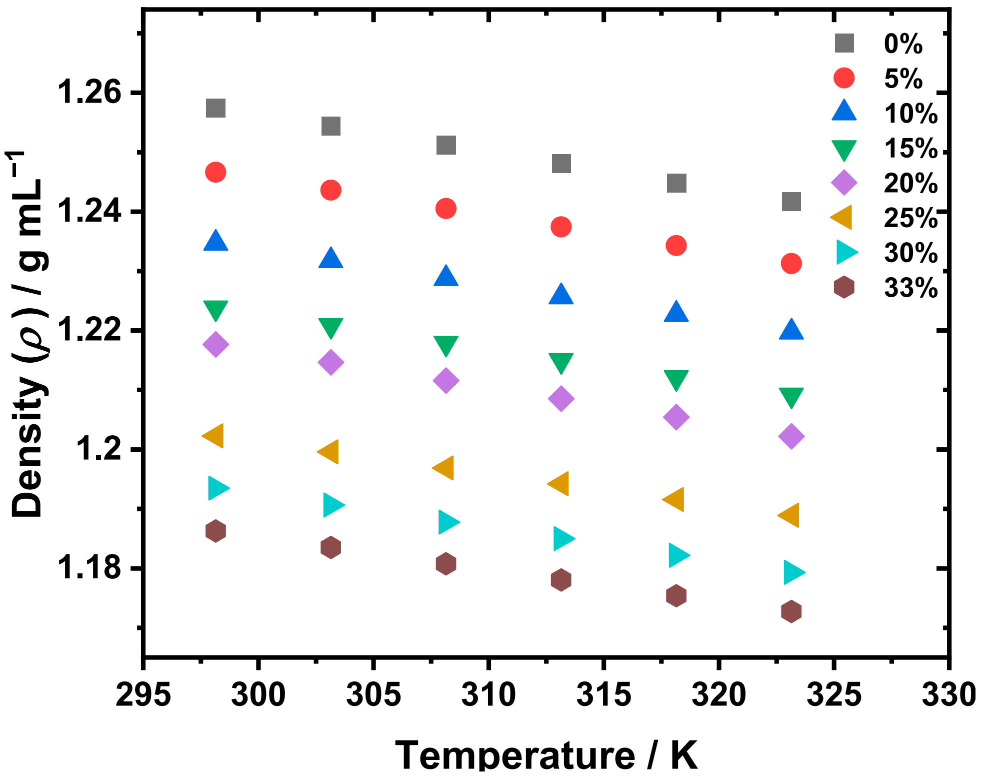

Figure S1. Density as a function of temperature, with legend representing varying mol % of ChCl in glycerol solutions. Density decreases as the temperature increases, which is shown in increments of 5 K, from 295.15 – 323.15 K. See Table S3 for numerical values and standard deviations. N = 3.
